# Integration of multi-omics data for prediction of phenotypic traits using random forest

**DOI:** 10.1186/s12859-016-1043-4

**Published:** 2016-06-06

**Authors:** Animesh Acharjee, Bjorn Kloosterman, Richard G. F. Visser, Chris Maliepaard

**Affiliations:** Wageningen UR Plant Breeding, Wageningen University & Research Centre, PO Box 6700 AJ, Wageningen, The Netherlands; Keygene NV, PO Box 216, 6700 AE, Wageningen, The Netherlands; MRC Human Nutrition Research, 120 Fulbourn Road, Cambridge, CB1 9NL UK

**Keywords:** Data integration, Genetical genomics, Networks, Random forest

## Abstract

**Background:**

In order to find genetic and metabolic pathways related to phenotypic traits of interest, we analyzed gene expression data, metabolite data obtained with GC-MS and LC-MS, proteomics data and a selected set of tuber quality phenotypic data from a diploid segregating mapping population of potato. In this study we present an approach to integrate these ~ omics data sets for the purpose of predicting phenotypic traits. This gives us networks of relatively small sets of interrelated ~ omics variables that can predict, with higher accuracy, a quality trait of interest.

**Results:**

We used Random Forest regression for integrating multiple ~ omics data for prediction of four quality traits of potato: tuber flesh colour, DSC onset, tuber shape and enzymatic discoloration. For tuber flesh colour beta-carotene hydroxylase and zeaxanthin epoxidase were ranked first and forty-fourth respectively both of which have previously been associated with flesh colour in potato tubers. Combining all the significant genes, LC-peaks, GC-peaks and proteins, the variation explained was 75 %, only slightly more than what gene expression or LC-MS data explain by themselves which indicates that there are correlations among the variables across data sets. For tuber shape regressed on the gene expression, LC-MS, GC-MS and proteomics data sets separately, only gene expression data was found to explain significant variation. For DSC onset, we found 12 significant gene expression, 5 metabolite levels (GC) and 2 proteins that are associated with the trait. Using those 19 significant variables, the variation explained was 45 %. Expression QTL (eQTL) analyses showed many associations with genomic regions in chromosome 2 with also the highest explained variation compared to other chromosomes. Transcriptomics and metabolomics analysis on enzymatic discoloration after 5 min resulted in 420 significant genes and 8 significant LC metabolites, among which two were putatively identified as caffeoylquinic acid methyl ester and tyrosine.

**Conclusions:**

In this study, we made a strategy for selecting and integrating multiple ~ omics data using random forest method and selected representative individual peaks for networks based on eQTL, mQTL or pQTL information. Network analysis was done to interpret how a particular trait is associated with gene expression, metabolite and protein data.

**Electronic supplementary material:**

The online version of this article (doi:10.1186/s12859-016-1043-4) contains supplementary material, which is available to authorized users.

## Background

In order to understand how quantitative variation in phenotypic traits is related to the underlying genetic differences between plants, and to differences in gene expression, protein constitution and metabolic variability, an approach is needed in which the combined molecular signature of the plants is shown to be predictive for the phenotypic traits of interest [1–3]. We can use high-throughput ~ omics technologies, such as gene expression microarrays [4, 5], mass spectrometry (LC-MS and GC-MS) [6, 7] and protein chips [8, 9] to obtain molecular signatures of a population of plants. In addition we can study phenotypic differences in the same population and hypothesize that differences in the phenotypic trait in the population are related to the variation in these combined molecular profiles across the different data sets [10]. Finding the ~ omics variables that are related to the phenotypic traits of interest can then be used in two ways: 1) for prediction of the traits from these molecular profiles. For example Steinfath et al., 2010 [11] explored metabolomics data to predict agronomic phenotypes of potato crop plants grown in different environments. They identified metabolites that can be used as biomarkers for these traits and hence to improve the selection on these traits which can be implemented in breeding programs. 2) to identify functional relationships between traits and molecular networks of the plants [11–12].

This means that we are not just interested in interrelating these ~ omics data sets for their own sake to find genetic and metabolic networks, but the networks and their elements should actually be predictive for a phenotypic trait of interest. On the other hand, in the context of genetics and plant breeding, we also want to be protected against finding relationships between phenotype and ~ omics data that are just caused by environmental or developmental differences. Instead, we are interested to find relationships that have a basis in the genetic differences between plants. Therefore a mapping population is an ideal target for this kind of study: we can study whether an observed relationship between a phenotypic trait and ~ omics variables is also based on genetic differences between the plants in the segregating population, as we can actually map the phenotypic variation as well as the variation in the ~ omics data sets. A relationship that would be just based on variation in environmental influences or conditions would not result in mapped QTLs for the phenotypic traits or the ~ omics variables [12, 13].

We are interested in the association between a number of phenotypic traits related to tuber quality of potato and several ~ omics data sets, some of which were published previously by us [14–16]. In addition, we use mapping and genotyping information since the population that we use is a mapping population. The quality traits considered are 1) potato tuber flesh colour, 2) enzymatic discoloration after peeling, 3) starch gelatinization as measured by differential scanning calorimetry (DSC) and 4) tuber shape. Out of these four phenotypes, starch gelatinization as measured by differential scanning calorimetry (DSC) and tuber shape phenotypes were not analyzed before. The other two phenotypes were included to make the link with the previous papers and to permit validation and accuracy testing of our used network analyses.

In this study, we relate transcriptomics, metabolomics and proteomics data to genetic variation in these quality traits in the mapping population. For this, we use a novel strategy consisting of three steps. First, using the same approach as in an earlier study [14], we apply Random Forest regression (RF) to find, per single trait and per individual ~ omics data set, the variables that play a significant role in the prediction of each of the quality traits. In the second step, we use QTL mapping of the quality traits and of the ~ omics variables to select variables that have a QTL (eQTL, mQTL or pQTL) cosegregating with a quality trait QTL and to remove redundancy in the set of selected variables. Finally, we construct regularized partial correlation networks, for each of the quality traits, of the selected sets of variables over the four ~ omics data sets that have a genetic association with the traits per QTL.

Because already much is known about the regulatory genetic and metabolic pathways involved in tuber flesh colour [17–19], we used this trait to validate the approach, as we had done for individual data sets in [14, 16, 19]. Here we analyze all ~ omics data sets simultaneously to construct networks related to phenotypic traits consisting of features across all these data sets. We demonstrate that also for the other phenotypic traits we can find networks of small sets of interrelated gene expression profiles, proteins and metabolites that are associated with and predictive for these quality traits.

## Methods

### Plant material

We used 96 individuals, including the parental clones, of a diploid potato backcross population (CxE) [20]. This population is derived from an original cross between potato clones C (USW533.7) and E (77.2102.37) and is described in detail in [21]. All clones were grown in multi-year repeats in the field, Wageningen, The Netherlands during the normal potato-growing season in The Netherlands (April–September) [14, 20, 21]. For each genotype, tubers were collected from three plants and representative samples were either used for phenotypic analysis or mechanically peeled and immediately frozen in liquid nitrogen before being ground into a fine powder and stored at −80 °C for metabolomics, transcriptomic and proteomic analyses. The determination of carotenoids was as described in [22]. This was a targeted metabolic analysis which includes compounds like zeaxanthin, violaxanthin and a compound for which the chemical behaviour is like violaxanthin (here denoted as ‘violaxanthin-like’).

Differential scanning calorimetry (DSC) is extensively used to study physical properties of starch granules. DSC-onset measurements (DSC onset) report water temperatures at which starch granules reach their gelatinization state [23]. DSC measurements provided gelatinization onset temperatures of starch granules for 96 individuals of the C x E population ranging between 61.5 °C and 66.7 °C. Enzymatic discoloration of tubers after peeling and being exposed to air (room temperature) at different time points such as after 5 min, 30 min, 3 h and difference in discoloration between 3 h and 30 min and 3 h is described in [18]. In this study, we considered only enzymatic discoloration after 5 min since the measurements at later time points are all highly correlated. Potato tuber shape was scored between 1 (round) and 5 (long) [21].

### Omics data sets

We generated different types of ~ omics data to integrate and find novel relationships among them. Parts of the ~ omics data sets were already published individually, for example, the transcriptomics data [14, 22], GC-MS data [16], LC-MS data [14]. Only the proteomics data was generated more recently (manuscript in preparation) and these data have not been considered for a combined analysis over all ~ omics technologies simultaneously.

In this section, we briefly summarize the different omics data generation methods and refer to [14, 16, 19, 22] for further details. The phenotypic data and ~ omics data are provided in a supplementary file (Additional file 1).

### Transcriptomic data

RNA was extracted from the 96 samples using the hot phenol method described previously [24]. (All samples were labeled with both Cy3 and Cy5-dye using the low RNA input linear Amplification Kit, PLUS, Two colour (Agilent technologies) according to the manufacturer’s protocol starting with 2 μg of purified total RNA [19]). For additional data analyses only genes with a Pearson correlation coefficient higher than 0.8 between the Cy3 and Cy5 datasets were included resulting in 15,062 expressed genes. We took into account only the Cy3 gene expression signals for further statistical analysis. For visualization, we used the gene nomenclature in the following way: Gene_Gene ID (for example: Gene_13945). The number refers to the gene ID of the supplementary material of [19].

### LC-MS, data generation, processing and identification

Potato tuber samples were analyzed for variation in semi-polar metabolite composition using an untargeted accurate mass LC-MS approach. In total 14,428 mass signals were obtained from mass spectrometry and selected based on the following criteria: signals should be present in at least 10 samples and with at least one amplitude higher than 100 units (about 5 times the noise value). Finally, data redundancy of signals derived from the same compound, i.e. isotopes, adducts and in-source fragments, was removed by by retention time-dependent clustering. This clustering of the 3024 signals resulted in 233 reconstructed metabolites (centrotypes). The untargeted metabolites are represented as centrotype_mass_scan number as in [14]. As an example: 818_795_918 means that the centrotype number is 818, the mass number 795 and the scan number 918. For visualization and simplicity we used LC-centrotype number (for example: LC_818). For more information on data generation, processing and identification see the Methods of [14].

### GC-MS data generation, processing and identification

GC-MS data were generated from the same 96 genotypes of the CxE population. Detailed materials and methods for GC-MS data generation, processing and identification are as in [16]. In short, raw data were processed by ChromaTOF software 2.0 (Leco Instruments) and MassLynx software (Waters), and further analysis was performed using MetAlign software to extract and align the mass signals. Mass signals that were present in fewer than two samples were discarded. Signal redundancy per metabolite was removed by means of clustering, and mass spectra were reconstructed [25]. This resulted in 139 reconstructed polar metabolites (representative masses called as “centrotype”). Compounds were subjected to tentative identification by matching to the NIST08 and Wiley spectral libraries. Library hits were manually curated, and a series of commercial standards was used to check annotation.

For visualization and simplicity we used GC-centrotype number. For example, 7275_20721700_73 means that the centrotype number is 7275, the mass number 20721700 and the scan number 73. For visualization only GC_7275 is used.

### Proteomics data generation, processing and identification

#### Protein extraction

Total protein was extracted from approximately 0.5 g of ground tuber material, to which 1 ml of pre-heated (95 °C) lysis buffer (50 mM sodium phosphate buffer pH 7, sucrose (5 % w/v), SDS (4 % w/v), DTT (0.3 % w/v), PVP-P (10 % w/v)) was added. Samples were homogenized and protein amount was measured using the RC/DC assay (Biorad, Veenendaal, the Netherlands).

#### Protein labelling

A single lysine per protein molecule was labelled using the fluorescent CyDyes from the Difference Gel Electrophoresis (DIGE) technology (GE Healthcare/Amersham Biosciences) according to the manufacturer’s protocol. The internal standard was labelled with Cy2 and consists of an equal mixture of 20 randomly chosen samples of the experiment (9 random samples from 2002 to 2003 each and both parents C and E from 2003).

Every 2D-gel contains one sample labelled with Cy3, one labelled with Cy5 and the internal standard labelled with Cy2. This means that every sample on each gel can be compared by using the internal standard sample labelled with Cy2.

#### 2D-Electrophoresis

Electrophoresis was performed using first dimension electrophoresis on 24 cm immobilized pH gradient strips (GE Healthcare/Amersham Biosciences) with a linear pH range from 4 to 7 on an Ettan IPGPhor isoelectric focusing (IEF) system. Cydye labelled samples (total of 150 μg protein) were loaded to the strips and focusing was run for 18 h at 20 °C with the following settings: 3 h 150 V, 3 h 300 V, from 300 V to 1000 V in 6 h, from 1000 V to 10,000 V in 1 h and finally 5 h at 10,000 V. After equilibration in the dark at room temperature in equilibration buffer (urea 6 M, 50 mM Tris–HCl pH 8.8, glycerol 30 % (v/v), SDS 2 % (w/v)) containing DTT 1 % (w/v)the second dimension electrophoresis was run on the Ettan Dalt 12 system on precast 12.5 % SDS polyacrylamide slab gel (size: 255x196x1 mm) and buffers from GE Healthcare/Amersham Biosciences. The separated CyDye-labelled proteins were visualized by scanning with a Ettan Dige Imager (GE Healthcare/Amersham Biosciences), using for Cy2 an 480 nm laser and an emission filter of 530 nm, for Cy3 an 540 nm laser and an emission filter of 595 nm and for Cy5 an 635 nm laser and an emission filter of 680 nm.

Gel images were analysed with the Decyder software version 7 according to Decyder 2Dv7 manual; GE Healthcare/Amersham Biosciences. Detected spots were filtered based on spot volume larger than relative value 30,000 to exclude spots that could be just background noise or dust particles. The internal standard in each gel was used to automatically match all images to the reference (the gel with the largest number of detected spots. The spot volume ratio to the internal standard of each protein and the individual volume of the spots were calculated and log_10_ transformed. In the QTL analysis the spot volume (intensity) value was used. Each of the proteins is presented by Pro_X where “X” represents consecutive protein numbers.

#### Protein identification

Spots of interest were excised from gel using the Ettan Spot Picker. Peptide mass determinations were carried out using the Applied Biosystems 4800 Proteomics Analyzer. Both PMF and MS/MS in reflectron mode analyses were carried out with the samples. Calibration was carried out with a peptide mass calibration kit. Proteins were identified by searching against the NCBI ‘viridiplantae’ and an EST ‘viridiplantae-eudicots’ database using MASCOT.

### Random Forest regression

Random Forest [26, 27] was used for regression of the phenotypic trait such as flesh colour, tuber shape, DSC onset, enzymatic discoloration on the transcriptomics, GC-TOF-MS signals, LC-MS signals, 2D-DIGE proteomics. RF constructs a predictive model for the response using all predictors but quantifies the importance of each, here the metabolites, gene expression and proteins in explaining the variation present in the trait. RF by itself does not provide significance levels of individual metabolites and does not perform a variable selection to choose a predictive subset of associated metabolites. Therefore, we included a permutation test to indicate significance of the association of a metabolite with a trait. In each of 1000 permutations of the trait values we estimated the variance explained by the RF model (R^2^) and the variable importance of each metabolite in terms of the decrease in node impurities [26]. We ordered node purity values from the permuted data sets and took the 95 percentile from the distribution of impurity values as the significance threshold of the individual metabolites. The same procedure was done for R^2^ values of the model: The 95 percentile was taken as a significance threshold for the RF model. R^2^ in Random Forest is not just a measure of goodness-of-fit of the data at hand but is determined on left-out samples (the ‘out-of-bag’ samples) so it should be interpreted as a measure for predictive quality (here considered as prediction R^2^) of the Random Forest on independent samples that have the same properties as the in-bag sample [26]. Random Forest analysis was done using R statistical software using the randomForest package.

### QTL analysis and cis- and trans-eQTLs

We mapped expression QTLs (eQTLs) from the gene expression data, metabolite QTLs (mQTLs) both for LC-MS and GC-MS, and protein QTLs (pQTL) from the proteomics data to find regions on the genome explaining genetic variation in gene expression, metabolite and protein values using the integrated linkage map of the C and E parents for QTL analysis [19].

Further, we used the potato genome physical map [28] to investigate eQTL physical positions of genes identified as predictive for phenotypic traits from the Random Forest analyses. The potato oligo (60-mer) microarray (POCI) used in the experiments contains 42,034 features based on a potato unigene set [15]. To allow discrimination between cis- and trans-eQTLs all unigenes were blasted against the genome scaffold sequences, predicted coding sequences (CDS) and predicted gene regions (including 5’ and 3’-UTR’s). Features with a unique and significant hit were assigned to genome scaffolds for which the majority has chromosome information. Identified QTLs on the same linkage group as their physical map position are identified as cis-acting while QTLs on different linkage groups are defined as trans-acting. Features on the array for which no physical map position could be assigned are classified as unknown [19]. All QTL analyses were done using metanetwork [29] package in R.

### Network reconstruction

From the RF analyses, for each of the traits, we obtain a list of significant genes, metabolites and proteins. For visualization in an association network, we reduced the number of genes by checking chromosomal positions of each of the predicted significant genes, metabolites (LC-MS and GC-MS) and proteins. We took the most significant single gene, LC-MS signal, GC-MS signal and/or protein per chromosome as representative for that ~ omics data set and chromosome and made a full order regularized partial correlation network with these genes, metabolites, proteins and traits as nodes in the network [30] and the strength of the interaction as edges in the network. Partial correlation measures the correlation between two variables after their linear dependence on other variables is removed. It can distinguish between direct and indirect associations whereas correlation-based network cannot and often yield many spurious edges [32–31]. A detailed description of regularized partial correlation is provided in [33].

## Results

### Selection of ~ omics features predictive for quality traits

The prediction of variation in potato tuber flesh colour [14] from the gene expression, LC-MS, and protein data sets was quite high (>50 % explained variance using all features, 60 to 75 % for smaller subsets of only significant features), but much lower for the GC-MS data (10 % and 33 % for unselected and selected features (Tables 1 and 2). For flesh colour, the microarray data and the LC-MS data were equally good for prediction in terms of the explained variance, but the numbers of significant features were very different: the prediction of flesh colour using 7 significant LC-MS features is almost the same as for 233 significant gene expression profiles (of which the genes are distributed over different regions in the genome) from the microarray data set. From the gene expression data, the gene which ranks first and third with respect to variable importance for predicting flesh colour was a beta-carotene hydroxylase (Bch). Two oligos were present on the array targeting the same beta-carotene hydroxylase gene, hence the two high ranks for the same gene. Another gene from the carotenoid pathway, zeaxanthin epoxidase (Zep) ranked forty-fourth. Based on our current knowledge of potato tuber flesh colour and carotenoid content [17, 22], these two genes were expected to be associated with flesh colour. From the GC-MS data, out of six significant metabolites, four had an annotation: malic acid, 2-4-5-trihydroxypentanoic acid, glucopyranose, 2-butenedioic acid(z)- and bis(trimethylsilyl) ester.

**Table 1:** Percentage variance explained (R^2^) in out-of-bag (OOB) prediction by Random Forest (RF) models using all genes, LC-peaks, GC-peaks or proteins separately

Quality trait	Gene expression	LC peaks	GC peaks	Proteins
Flesh colour	58 %	63 %	10 %	53 %
Tuber shape	32 %	NS	NS	NS
DSC Onset	42 %	NS	12 %	22 %
Enzymatic discoloration	14 %	16 %	NS	13 %

Non-significant models are indicated as NS ( alpha  = 0.001)

**Table 2:** Percentage variance explained (R^2^) using only significant gene expression, LC-peaks, GC-peaks or proteins

Quality trait	Gene expression	LC peaks	GC peaks	Proteins	Combining significant genes, LC peaks, GC peaks and proteins	Combining significant genes, LC peaks, GC peaks and proteins from max. one QTL per dataset per chromosome
Flesh colour	73 % (233)	74 % (7)	33 % (6)	60 % (10)	75 % (256)	73 % (Fig. 1) Gene(8) + LC(2) + GC(2) + Protein(1); Total =13
Tuber shape	55 % (303)	NS	NS	NS	55 % (303)	53 % (Fig. 2) Gene (11)
DSC onset	44 % (487)	NS	27 % (5)	28 % (2)	51 % (494)	45 % (Fig. 3) Gene(12) + GC(5) + Protein(2); Total = 19
Enzymatic discoloration	51 % (420)	32 % (8)	NS	36 % (22)	46 % (450)	43 % (Fig. 4) Gene(4) + LC(2) + Protein(2); Total = 8

The numbers of significant (alpha=  0.001) genes, LC-peaks, GC-peaks or proteins are between brackets. Non-significant models are indicated as NS

For the three other traits the gene expression data was more predictive than the metabolomics and proteomics data sets, and for tuber shape actually no significant features were found for GC-MS, LC-MS and the protein data, while significant gene expression differences explain 55 % of the variation in tuber shape. For starch gelatinization (DSC) no LC-MS features were significant, for enzymatic discoloration no GC-MS features were significant.

### Associated genomic regions

Using Random Forest regression, we generated lists of candidate genes, metabolite features and proteins that are predictive for quality traits of potato tubers (Tables 1 and 2). However, from this prediction we cannot conclude that these associations necessarily have a basis in genetic differences between the plants since they could also be caused by environmental variation in both the trait and the levels of the features, or in developmental differences. Therefore we also investigated QTL positions for both the phenotypic traits and the ~ omics features. When co-segregation of an eQTL, mQTL or pQTL with a trait QTL is observed, this could imply a functional relationship or identify causal genes/proteins/metabolites. However, this is not necessarily the case since any linked but functionally unrelated QTLs will also show this co-segregation. Still, these sets of predictive genes highlight genomic regions of interest, comparable to standard QTL analysis, and can provide additional information or help to narrow down the region of the causative polymorphism.

For tuber flesh colour the 233 eQTLs of significantly predictive gene expression profiles from RF mapped to eight chromosomes: 2, 3, 4, 5, 8, 9, 11 and 12. A large number of these (132) eQTLs were mapped to chromosomes 2 and 3. Significant GC-MS peaks mapped to two chromosomes, 1 and 2. QTLs for significant LC-MS peaks mapped to chromosomes 2 and 3 and QTLs for significant protein spots mapped to chromosome 3. Using 13 variables (one per chromosome with a QTL, per data set) as predictor set for flesh colour the RF out-of-bag prediction explains 73 % of the phenotypic variation.

For tuber shape the expression QTLs of the significant genes mapped to chromosome 1 to 11 but not to chromosome 12. However, the largest number (185) expression QTLs of the significant genes map to chromosome 10. Using one representative gene from each of those 11 chromosomes as a predictor set for tuber shape, the RF prediction explains 53 % of the variation in the trait. No proteins or GC- or LC-variables were included since none were significantly associated to tuber shape. Only the chromosome 10 representative gene explains already 38 % of the phenotypic variation.

For starch gelatinization (DSC onset), the eQTLs of the significant genes mapped to all the 12 chromosomes but the largest number (201) mapped to chromosome 2. QTLs for significant GC-MS peaks mapped to five chromosomes: 2, 4, 5, 9 and 11. QTLs for significant proteins were mapped to chromosomes 2 and 5. These 19 selected variables explain 45 % of the variation in starch gelatinization.

For enzymatic discoloration the expression QTLs of the significant genes mapped to four chromosomes: 1,3,5 and 8. QTLs for significant LC-MS peaks mapped to chromosomes 3 and 5 and for significant protein spots to chromosomes 1 and 3. These 8 selected variables (genes and LC-MS peaks) explain 43 % of the variation in enzymatic discoloration.

### Integration of ~ omics data and network visualization

**Fig. 1:**
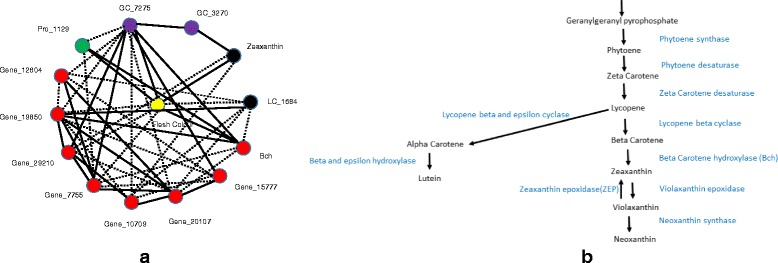
(**a**) A partial correlation network of the phenotypic trait tuber flesh colour (yellow) with gene expression features (red), metabolites from LC-MS (black), metabolites from GC-MS (purple) and proteins (green). The dotted lines represent negative partial correlation coefficients, solid lines represent positive partial correlation coefficients. Bch = beta-carotene hydroxylase, LC_X represents metabolites derived from LC-MS with centrotype number X, GC_X represents metabolites derived from GC-MS with centrotype X, Gene_X = Gene with gene ID X. Pro_X represents a protein with protein ID X. (**b**) Shows the existing published part of the carotenoid pathway [37], and some of the genes: Bch and Zep are identified by our data

**Fig. 2:**
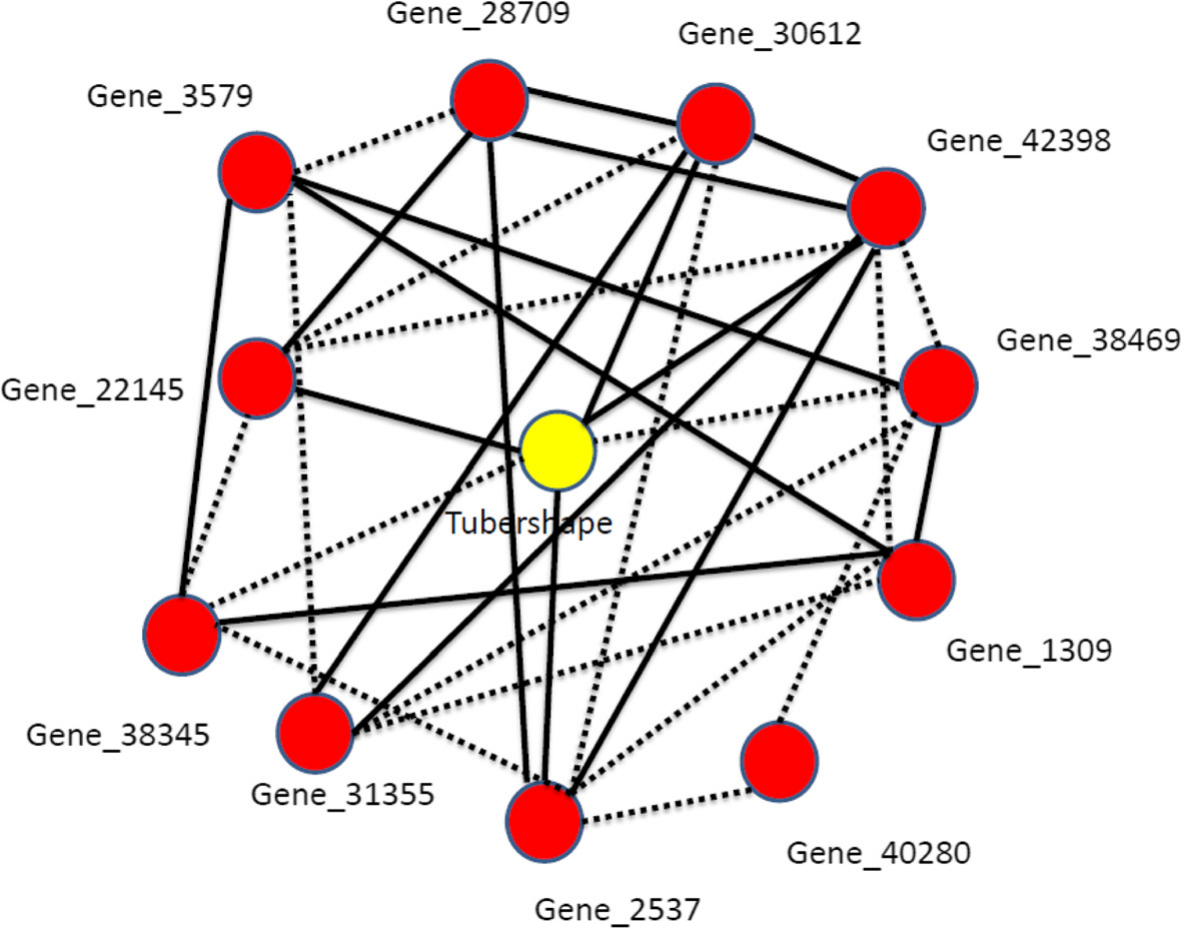
A partial correlation network of tuber shape (yellow) with gene expression features (red). The dotted lines represent negative partial correlation coefficients, solid lines represent positive partial correlation coefficients. Gene_X = Gene with gene ID X

**Fig. 3:**
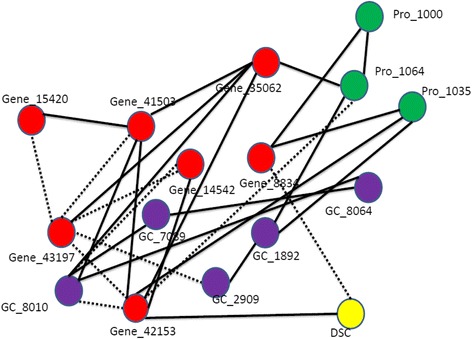
A partial correlation network of DSC onset (yellow) with gene expression features (red), metabolites from GC-MS (purple) and proteins (green). The dotted lines represent negative partial correlation coefficients, solid lines represent positive partial correlation coefficients. GC_X represents metabolites derived from GC-MS with centrotype X, Gene_X = Gene with gene ID X. Pro_X represents proteins with protein ID X

**Fig. 4:**
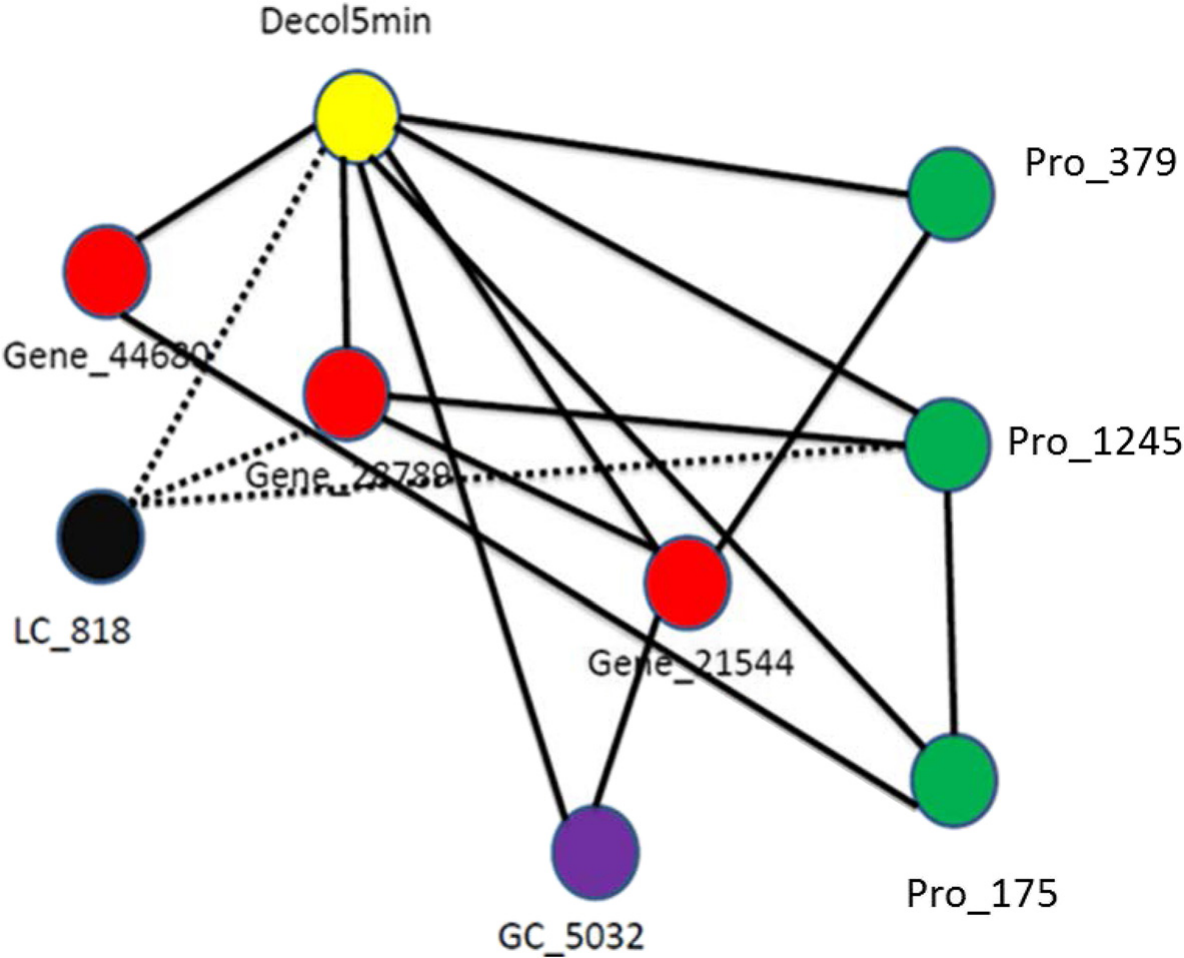
A partial correlation network of enzymatic discoloration (yellow) with gene expression features (red), metabolites from LC-MS (black) and proteins (green). The dotted lines represent negative partial correlation coefficients, solid lines represent positive partial correlation coefficients. LC_X represents metabolites derived from LC-MS with centrotype number X, Gene_X = Gene with gene ID X. Pro_X represents proteins with protein ID X

With Random Forest we selected genes, metabolites and proteins that have a significant association with quality traits. Many of these have QTLs on the same genomic regions and can be considered as redundant. Therefore we selected a single representative feature per QTL region per data set for network visualization (Figs. 1, 2, 3 and 4). The networks show partial correlations with a lasso penalty. The nodes in the network show the phenotypic trait of interest and selected genes, metabolites and proteins (one per QTL per data set). Positive partial correlations are shown in solid lines, negative partial correlations in dotted lines.

Networks reconstructed from the integrated ~ omics technology platforms can provide a formal framework for investigating plant metabolism. For example, the network shown in Fig. 1a is completely based on the ~ omics data, the one in Fig. 1b is based on the known carotenoid pathway as in [34]. In Fig. 1b we can see that Bch interacts with zeaxanthin and that violoxanthin is produced. By using only the ~ omics data (Fig. 1a) we could reproduce part of the carotenoid pathway. This finding is extended to other traits as well in order to hypothesize how genes/proteins/metabolites are regulated.

## Discussion

We used Random Forest regression for integrating transcriptomics, metabolomics and proteomics data for prediction of four quality traits of potato: tuber flesh colour, DSC onset, tuber shape and enzymatic discoloration. For each of these traits, we selected sets of genes, metabolites and proteins that were significant in explaining variation in the trait. We then quantified the amount of variance explained in the prediction using these selected sets of ~ omics features, and we constructed partial correlation networks for subsets of genes, metabolites and proteins using QTL mapping information.

### Flesh colour

For validation of our approach, we took advantage of what was known about the relationship between the trait tuber flesh colour and previously published genes associated to flesh colour. In our analyses, we found that beta-carotene hydroxylase [35] and zeaxanthin epoxidase (Zep) [17] were ranked first and forty-fourth respectively for prediction of flesh colour and both compounds have previously been associated with flesh colour in potato tubers [35].

In addition, for the GC-MS data we found that six metabolites were significant and out of these six, four were annotated. It was observed that one of the compounds was identified as malic acid. Although there is no direct link between tuber flesh colour and malic acid, [36] reported a correlation of malic acid to skin colour in apricot. For the other three metabolites we did not find any connection with carotenoids or with flesh colour in the literature.

Using all the significant genes across the technology platforms, the combined LC-peaks, GC-peaks and proteins, the OOB variation explained (R^2^) was 75 %, only slightly higher than what gene expression or LC-MS data explain by themselves (Table 2) which indicates that there are correlations among these variables across the data sets. This is indeed the case: for example, from the proteomics data, Pro_1129 and 1684_644_1727 from the LC-MS data are strongly correlated with beta-carotene hydroxylase gene expression, with Pearson correlation coefficients of −0.73 and 0.78 (Additional file 2) and, partial correlations of −0.66 and 0.52, respectively. Based on the correlations among the selected ~ omics data, two main clusters of ~ omics features related to flesh colour are visible (Additional file 2).

### Tuber shape

For tuber shape regressed on the gene expression, LC-MS, GC-MS and proteomics data sets separately, only gene expression data was found to explain significant variation. More than half of the eQTLs of significant genes are mapped to chromosome 10. Tuber shape is thought to be regulated by a single locus Ro on chromosome 10, where round (Ro) is dominant over long (roro) [37, 38]. At the Ro-locus a series of multiple alleles can explain all intermediate shapes between round (going to flat) and long [37]. The large number of eQTLs of genes predictive for tuber shape can be due to linkage to this Ro locus. No metabolites and proteins were found as significantly predictive of tuber shape, which indicates that for this particular trait, gene expression data is more informative than metabolomics or proteomics data.

### DSC onset

For DSC onset, we found 12 significant gene expression, 5 metabolite levels (GC) and 2 proteins that are associated with the trait. Using those 19 significant variables, the variation explained was 45 %. In order to find what genomic regions the selected genes are regulating, eQTL analyses were performed. The analyses showed many associations with genomic regions in chromosome 2 with also the highest explained variation compared to other chromosomes. For gene contig MICRO.9632.C4 the eQTL analysis revealed a large QTL region at the bottom of chromosome 2 between 73 cM and 86 cM. eQTL analysis for EST BF_LBCHXXXX_0013B11_T3M.SCF produced a single QTL on the same chromosomal location. For the GC-MS data, out of five metabolites we could identify three: proline, glucopyranose and 2-Piperidone that were associated to DSC onset. The two proteins that were associated with DSC onset could not be identified.

### Enzymatic discoloration

Transcriptomics and metabolomics analysis was previously done for enzymatic discoloration after 5 min and resulted in 420 significant genes and 8 significant LC metabolites, among which two were putatively identified as caffeoylquinic acid methyl ester and tyrosine [14, 18].

In this paper we used genetic information through QTL analysis on the one hand and prediction of the traits using RF analysis from transcriptomics, metabolomics and proteomics analysis on the other hand. From the QTL analyses, we can identify the map position of the QTLs for gene expression or metabolite signals but we expect that functional genes and genes that are only linked and that influence other pathways will show similar correlations. For example: we obtain a large number of significant genes for prediction of flesh colour mapped to chromosome 3 but from previous published results [14, 35], we know that only Bch on chromosome 3 is responsible for flesh colour, so it is likely that the remaining genes are all associated because of linkage to Bch.

In RF regression, the prediction of phenotype from metabolomics, transcriptomics or proteomics data is possible in a way that genes, metabolites and/or proteins might be linked with a phenotype but independent of the genetic information [14]. In this study where we combine the prediction of the phenotype with QTL information, the selected genes are not only significant for predicting the phenotype of interest but also co-localized with the phenotypic trait QTLs. Further, for the predictive genes we checked their location in the genome sequence to find genomic positions of those genes irrespective of QTL analysis.

The key advantage of eQTL, mQTL or pQTL mapping in addition to the traditional mapping of phenotypic QTLs, is that it connects variation at the level of RNA expression, metabolite or protein abundance to variation at the level of DNA. The latter provides versatile tools for breeding whereas the first can reveal information on the biology of a trait and can direct to new candidate genes. Mapping of eQTL to the gene itself indicates that cis-regulation is responsible for the different expression levels, whereas map positions of eQTL different from the position of the corresponding genes indicate trans-regulation which allows deriving regulatory networks of genes [22].

In this study, RF regression was used as a tool for data integration of metabolites, gene expression and protein profiles relating to a phenotypic trait of interest where it was used to identify leads for further exploration. For example: flesh colour was associated with metabolites and after putative identification of the metabolic peaks 4,7-Megastigmadiene-3,9-diol-glucoside and 2,3-Dihydroxy-4-megastigmen-9-one-glucoside were identified as carotenoid derived compounds. Such approaches give us leads for further research on the metabolites and help to hypothesize which components (genes, metabolites, proteins) are in a specific pathway of interest and the genetic basis of the genes, metabolites or proteins involved in the pathway. Metabolite peaks that are not identified but that also show an association to the trait, could in many cases be breakdown products of the carotenoid pathway [22].

## Conclusions

In this study, we devised and evaluated a strategy to integrate multiple ~ omics data with phenotypic traits of interest and to select sets of co-expressed genes, metabolites and proteins. Prediction of significant features was obtained through RF regression, then through a genetical genomics study [12] we mapped those QTLs from gene transcripts (eQTLs), metabolites (mQTLs) and proteins (pQTLs). We selected a single gene transcript, metabolite and/or protein per chromosome if multiple features mapped to the same position to use for an integrated network analysis and visualization using lasso regularized partial correlations; this network can subsequently be used as a predictive network for the trait of interest. By doing so, we selected genes, proteins and metabolites which are not only predictive for a trait of interest but also explain variation of the trait of interest. Further, we explored previously published results on the same mapping population and extended our integrative approach, previously applied to transcriptomics and metabolomics data sets [14, 16, 18] on the combined data over all the ~ omics platforms (transcriptomics, GC- and LC-MS and proteomics).

For the selected combined data sets as stated before the results are interesting because there is an improvement of the R^2^ prediction value as compared to the unselected data. This improvement is most likely due to filtering out noise variables from the data set, as is explained in [14] in more detail. For DSC onset, none of the LC-MS peaks were significant which might indicate that primary metabolism is more important for this trait compared to secondary metabolism, whereas for enzymatic discoloration the situation is the other way around.

Now questions arise to which type of ~ omics data is important to study a trait. Our study indicates that, depending on the type of trait, different data sets are important. For example, if the trait is a quality trait then metabolomics data will be useful to investigate further the biochemical pathways and to arrive at potential candidate genes (for example: flesh colour was associated with metabolites and after putative identification of the metabolic peaks 4,7-Megastigmadiene-3,9-diol-glucoside and 2,3-Dihydroxy-4-megastigmen-9-one-glucoside were identified as carotenoid derived compounds) [14].

We selected significant genes, metabolites and proteins based on permutation tests and finally selected representative individual peaks for networks based on eQTL, mQTL or pQTL information. Network analysis was done to interpret how a particular trait is associated with gene expression, metabolite and protein data. For these networks, we used regularized partial correlation coefficients because these quantify the correlation between two variables (e.g. gene and metabolite, gene and protein or metabolite and protein) while conditioning on one or several other variables [39]. Those genes, metabolites and proteins might be considered as leads with connections to the phenotype. Although the identified chromosomal regions do not lead automatically to genes or metabolites directly involved in the trait, and it might be necessary to know more about the metabolic pathway and indirect acting genes and/or metabolites the locations surely will help us to zoom in on potential candidate genes. In other words, even though we are not finding the genes directly, this procedure is still helpful in guiding us towards metabolic pathways or genetic regulation of these pathways. However, further study would be needed to analyze the combined effect of multiple QTL regions over different chromosomes.

An important limitation of this approach is validation in the absence of prior knowledge regarding genes, metabolites or proteins of the trait under investigation; a second limitation is the difficulty in identification of metabolites and especially proteins. Further, validation of such predictive networks is necessary to show that statistical association is also pointing to functional relationships in biology.

## Additional files

Additional file 1: List of the data sets used in this article. (XLSX 14739 kb) Additional file 2: Hierarchical clustering with the Pearson correlation. (DOCX 53 kb)

## Abbreviations

Bch: beta-carotene hydroxylase
Cy3: Cyanine 3
Cy5: Cyanine 5
DSC: differential scanning calorimetry
eQTL: expression quantitative trait loci
GC-MS: gas chromatography–mass spectrometry
LC-MS: liquid chromatography–mass spectrometry
mQTL: metabolic quantitative trait loci
OOB: out-of-bag error from Random Forest
pQTL: protein quantitative trait loci
QTL: quantitative trait loci
RF: Random Forest
Zep: zeaxanthin epoxidase
