# Supplementary material for: Integration of multi-omics data for prediction of phenotypic traits using random forest
Source: BMC Bioinformatics. 2016 Jun 6;17(Suppl 5):180. doi: 10.1186/s12859-016-1043-4 (PMC4905610; doi:10.1186/s12859-016-1043-4)
Supplement: Additional file 2: — Hierarchical clustering with the Pearson correlation. (DOCX 53 kb) [file 12859_2016_1043_MOESM2_ESM.docx]

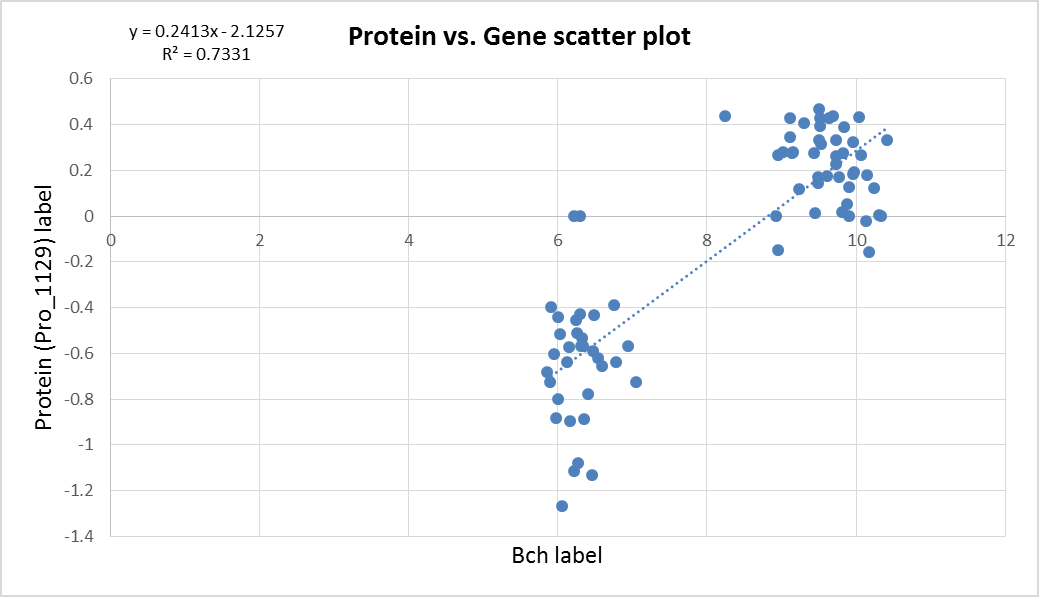


Figure 1 : Shows relationships with Bch and protein (pro_1129)


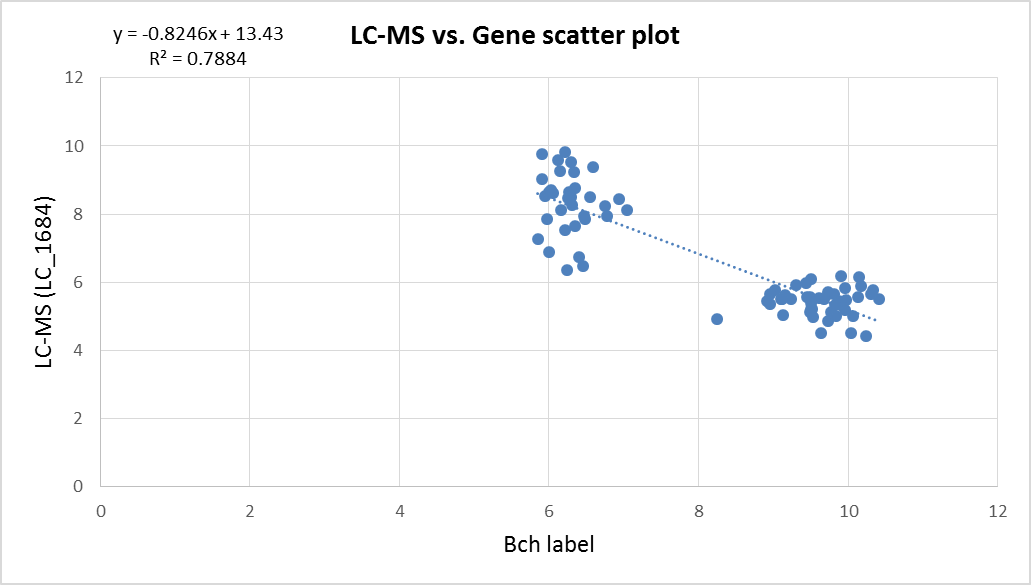


Figure 2: Shows relationships with Bch and LC-MS data (1684_644_1727)
